# Proteome Analysis of Date Palm (*Phoenix dactylifera* L.) under Severe Drought and Salt Stress

**DOI:** 10.1155/2016/7840759

**Published:** 2016-10-20

**Authors:** Haddad A. El Rabey, Abdulrahman L. Al-Malki, Khalid O. Abulnaja

**Affiliations:** ^1^Biochemistry Department, Faculty of Science, King Abdulaziz University, Jeddah, Saudi Arabia; ^2^Bioinformatics Department, Genetic Engineering and Biotechnology Institute, Sadat City University, Sadat City, Minufiya, Egypt

## Abstract

Date palm cultivars differently tolerate salinity and drought stress. This study was carried out to study the response of date palm to severe salinity and drought based on leaf proteome analysis. Eighteen-month-old date palm plants were subjected to severe salt (48 g/L NaCl) and drought (82.5 g/L PEG or no irrigation) conditions for one month. Using a protein 2D electrophoresis method, 55 protein spots were analyzed using mass spectrometry. ATP synthase CF1 alpha chains were significantly upregulated under all three stress conditions. Changes in the abundance of RubisCO activase and one of the RubisCO fragments were significant in the same spots only for salt stress and drought stress with no irrigation, and oxygen-evolving enhancer protein 2 was changed in different spots. Transketolase was significantly changed only in drought stress with PEG. The expression of salt and drought stress genes of the chosen protein spots was either overexpressed or downexpressed as revealed by the high or low protein abundance, respectively. In addition, all drought tolerance genes due to no irrigation were downregulated. In conclusion, the proteome analysis of date palm under salinity and drought conditions indicated that both salinity and drought tolerance genes were differentially expressed resulting in high or low protein abundance of the chosen protein spots as a result of exposure to drought and salinity stress condition.

## 1. Introduction

Plant species differ in their tolerance to abiotic stress. Salt sensitivity causes both rapid osmotic phase inhibiting growth of young leaves and a slower, ionic phase accelerating senescence of mature leaves causing reduction in crop plants yield [1]. Proteome analysis is a convenient tool for testing the response of plants to abiotic stress [2, 3]. Date palm (*Phoenix dactylifera* L.) can adapt to extreme drought, heat, and relatively high levels of soil salinity [4]. Date palms can grow under a variety of environmental conditions such as heat, and water shortage and salinity of the ground water provide abiotic stresses which decrease date production [5].

Plants respond to a stress by modulating abundance of candidate proteins, either by upregulating expression or by the synthesizing novel proteins primarily associated with plant defense system [6]. Therefore, proteome of plant species changes as a response to biotic “pests” [7–9] and abiotic “chemical, salt, and drought stress” [3, 10, 11]. Proteome analysis of roots and leaves revealed a synergetic responsive network under stress; roots rapidly sensed and responded to stress, after which the stress signals were transferred to leaves and both roots and leaves showed similar metabolic pathways under stress with distinct changes [12]. Under salinity, salt is transported via the xylem to the shoot causing the accumulation of Na^+^ and Cl^−^ ions in shoot cells to a toxic extent, resulting in ionic stress that leads to ion accumulation enhancing the production of reactive oxygen species (ROS). The extra production of ROS in cells leads to a disruption of the balance between production and removal of ROS which eventually leads to oxidative stress [13]. Paul et al. [14] stated that the major class of identified proteins resulting from salinity stress response belongs to carbohydrate and energy metabolism category while stress and defense related proteins are especially up-accumulated under drought stress and a novel protein, “R40C1”, was reported to be up-accumulated in roots of transgenic plants which may play an important role in generation of drought tolerant plants.

Recent proteome analyses have identified numerous drought-responsive proteins, which are involved in redox regulation, oxidative stress response, signal transduction, protein folding, secondary metabolism, and photosynthesis [15, 16]. Production of proline is a common response to various abiotic stresses and its differential accumulation cannot be used as a molecular marker in date palm breeding programs aimed at improving drought or salinity tolerance traits in date palms. This conclusion is consistent with the theory that the molecular outcomes of abiotic stresses are often nonspecific [17].

This study aimed to analyze proteome of eighteen-month-old date palm plants subjected to severe salt (48 g/L NaCl) and drought (82.5 g/L PEG or no irrigation) conditions for one month. Changes in proteins levels under salinity and drought stress helped in defining genes correlated with these stress conditions that encoded these proteins.

## 2. Materials and Methods

### 2.1. Stress Experiment

**Figure 1:**
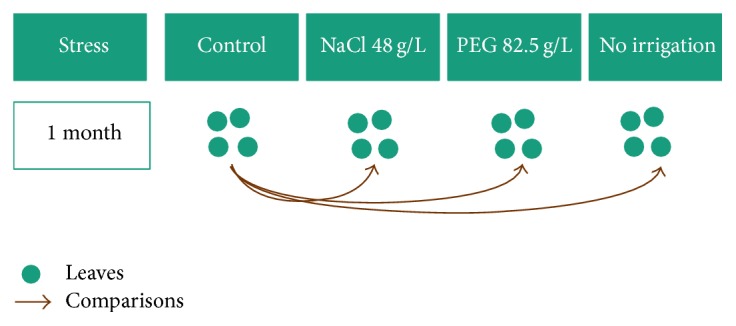
Scheme of the performed proteome analysis.

 Eighteen-month-old plants of date palm originated from tissue culture were subjected to three stress conditions for 30 days. The stress conditions were salt stress using 48 g/L NaCl, drought using 82.5 g/L, and drought due to no irrigation. The experiment was terminated, when nonirrigated samples started to wilt and the other treatments showed dwarfism and reduction in the leaf area compared to the control plants. Salt stress samples (76, 77, 78, and 80, see Table 1), 4 drought stress samples from PEG treatment (86, 88, 89, and 90), 4 drought stress samples with no irrigation (61, 62, 63, and 64), and 4 control samples at the end of the experiment (51, 52, 54, and 55) were chosen for proteome analysis as shown in Figure 1.

### 2.2. Protein Extraction, Labeling, and 2D Electrophoresis

Protein was extracted according to the method described in El Rabey et al. [11]. Briefly, four replicates of the frozen shoot of stressed and control plants were ground into a fine powder in liquid nitrogen. Proteins were precipitated by the addition of 1.8 mL of ice-cold acetone containing 0.07% (v/v) mercaptoethanol. One hundred mg of each sample was dissolved in 400–600 *μ*L of IEF buffer (7 M urea, 2 M thiourea, 2% (w/v) CHAPS, and 30 mM Tris, pH 8.0). The proteins were resolubilized overnight at room temperature. The mixture was then centrifuged for 10 minutes at 4°C at 16,100 ×g and total soluble protein in the supernatants was quantified using the 2D* Quant* Kit (GE Healthcare, Munich, Germany). Labeling and mixing scheme for the performed DIGE experiment is shown in Table 1. Fifty micrograms of total soluble protein was used for analysis of each sample in 2D gel. Eight analytical gels were run in pH range 4–7. Part of the internal standard was saved before labeling with CyDye 2 and this unlabeled part was applied to 2 preparative gels that were in the end used for protein identification by mass spectrometry. Proteins from the extracts were separated in the first dimension according to their pI using IEF on Immobiline DryStrip, 24 cm, pH 4–7, and in the second dimension according to their molecular weight using SDS-PAGE. All the 8 analytical gels and the 2 preparative gels were run at the same time.

After protein separation, the gels were scanned. Three fluorescence scans for each analytical gel were acquired and used for the analysis. The preparative gels were fixed for 1 hour in fixation solution (40% ethanol and 10% acetic acid in water) and then stained with RuBPS (1 mM in fixation solution) for 20 minutes and destained overnight in fixation solution. The preparative gels were scanned directly after destaining and stored wet at 4°C before spot cutting.

### 2.3. Biological Variation Analysis (BVA)

BVA allowed quantitative comparisons of protein expression across multiple gels.* t*-test (*p* value calculated using Student's* t*-test) and average ratio (fold difference in protein abundance of one protein spot between the groups selected in the protein statistics) values were calculated for all matched spots. The spots were filtered for* t*-test *p* value lower than 0.05 and Av. ratio > 2 and <−2. All automatically chosen spots were checked manually if they are real spots and marked for picking in the scan of the preparative gel.

### 2.4. Identification of Proteins in the Spots of Interest Chosen by DIGE Analysis

55 spots were cut from the preparative gel for MS analysis. Proteins in the gel plugs were reduced with 10 mM dithiothreitol and alkylated using 55 mM iodoacetamide in 0.1 M to open S-S bridges for action of trypsin. Digestion with trypsin (12.5 ng/*μ*L of trypsin in 50 mM NH_4_HCO_3_) was performed overnight at 37°C. The resulting peptides were extracted from the gel plugs in two extraction steps: first one with 25 mM NH_4_HCO_3_ and second one with 5% formic acid. Collected extracts were dried down and resolubilized in 2% acetonitrile with 0.1% formic acid in water (MS grade) for MS analysis. The resulting peptides were separated according to their hydrophobicity by nanoHPLC (C18 column, UltiMate* 3000 HPLC* system, Dionex) and sprayed directly into an ion trap spectrometer (amaZon ETD, Bruker Daltonics) using nanoESI sprayer.

### 2.5. Data Mining

Processed MS/MS spectra were used for the protein identification with in-house Mascot Search server (Matrix Science software, Matrix Science Ltd., London, UK). Swiss Prot (all species) and NCBInr (Green plants) databases were involved in the protein search.

## 3. Results

### 3.1. Results of 2D-Gel Electrophoresis

**Figure 2:**
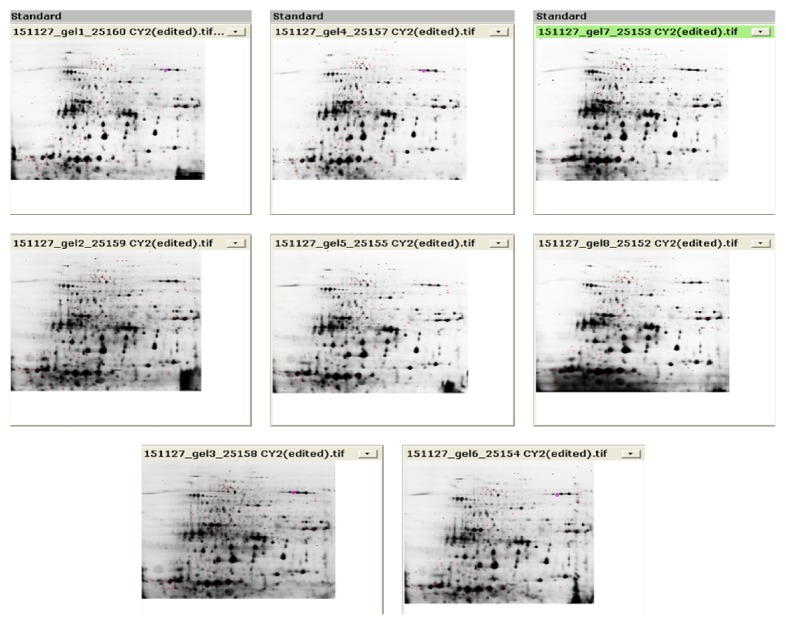
Overview of the fluorescence scans, internal standards.

**Figure 3:**
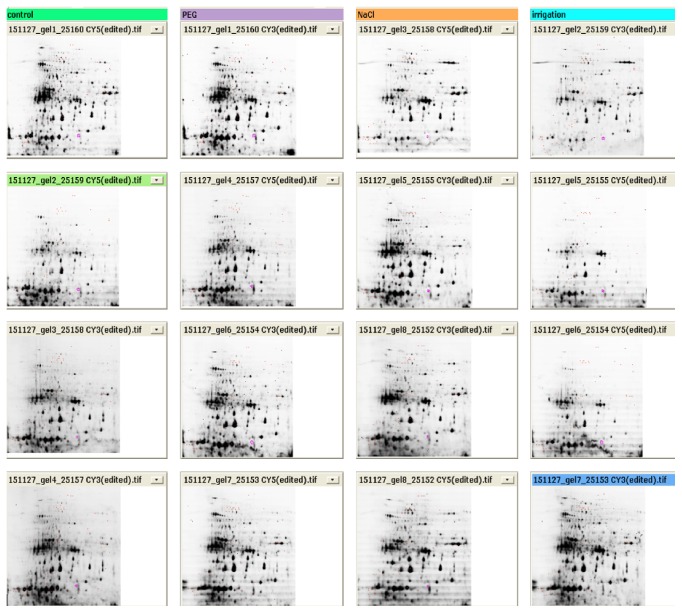
Overview of the fluorescence scans, analytical gels. Color code for analyzed samples: green, control; violet, PEG drought stress; orange, salt stress; blue, no irrigation drought stress.

**Figure 4:**
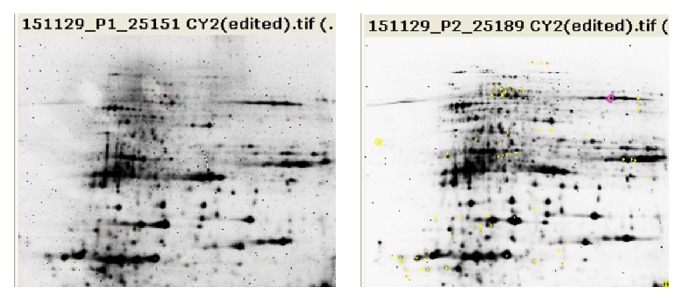
Overview of the fluorescence scans, preparative gels.

Figures 2, 3, and 4 show the three fluorescence scans for each analytical gel used for the analysis, whereas Table 2 shows the results of filtered spots by* t*-test *p* value lower than 0.05 and Av. ratio > 2 and <−2. Supplementary Figure  5, in Supplementary Material available online at http://dx.doi.org/10.1155/2016/7840759, shows location of the automatically chosen spots that were checked manually if they are real spots and marked for picking in the scan of the preparative gel.

### 3.2. DIGE Analysis

Table 2 illustrates detailed results of the 55 protein spots that were significantly changed as a result of salinity and drought stress as revealed by DIGE analysis. The results can be illustrated as follows: (i) in the salt stress plants, 15 spots showed higher protein abundance and 20 spots showed lower protein abundance in the leaves of the stressed plants compared to control plants of the same age; (ii) the drought stress with PEG produced three spots with higher protein abundance and 6 spots with lower protein abundance in the leaves of the stressed plants compared to control plants of the same age; (iii) drought stress with no irrigation did not produce any spots with higher protein abundance in spite of producing 19 spots with lower protein abundance in the leaves of the stressed plants compared to control plants of the same age; (iv) six spots showed lower protein abundance in two studied stresses, salt stress and drought stress due to no irrigation; (v) only 1 spot showed lower protein abundance in all three studied stresses.

The 55 spots of Table 2 were chosen for protein identification by mass spectrometry, bigger portion of them with lower protein abundance under stress conditions compared to the unstressed control as revealed above.

### 3.3. MS Analysis and Data Mining

The results of MS analysis of the chosen 55 spots showed that 42 spots out of them were analyzed, whereas the other 13 were not analyzed due to no protein hit or not analyzed by MS (see the Supplemented Excel File). A BLAST-p search of the resulting protein sequence was done using NCBInr database/Green plants database for homologous protein search.

The spots with higher protein abundance in PEG treated group (which are listed in Table 2) were ribulose-1,5-bisphosphate carboxylase/oxygenase (RubisCO) large subunit [*Zantedeschia aethiopica*] and transketolase (TRK), chloroplastic [*Phoenix dactylifera*], whereas those with high protein abundance in the NaCl treated group were uncharacterized protein LOC103705614 [*Phoenix dactylifera*], chaperonin hsp60 [*Arabidopsis thaliana*], chaperonin CPN60-2, mitochondrial-like [*Phoenix dactylifera*], serine acetyltransferase 1, chloroplastic-like [*Brassica napus*], beta-glucosidase 12-like [*Phoenix dactylifera*], ATP synthase CF1 alpha chain (ATPase) [*Phoenix dactylifera*], ATP synthase subunit beta, mitochondrial-like [*Phoenix dactylifera*], ATP synthase beta subunit [*Toronia toru*], serine acetyltransferase 1 (SAT1), chloroplastic-like [*Brassica napus*], and ribulose-1,5-bisphosphate carboxylase.

The distribution of the MS analyzed 42 spots that were significantly changed (*p* < 0.05) as a result of salinity and drought stress using either PEG or no irrigation compared to the control is as follows:

- Four proteins were significantly changed in all studied salinity and drought treatments (oxygen-evolving enhancer protein 1 (OEE1), chloroplastic [*Phoenix dactylifera*], elongation factor TuA (EFTuA), chloroplastic-like [*Phoenix dactylifera*], ATP synthase CF1 alpha chain [*Phoenix dactylifera*], and ribulose-1,5-bisphosphate carboxylase/oxygenase large subunit [*Villarsia albiflora*]).
- Nine proteins were changed in both no irrigation and NaCl treated groups (ribulose-1,5-bisphosphate carboxylase/oxygenase large subunit, partial (chloroplast) [*Ulva lactuca*], ribulose bisphosphate carboxylase/oxygenase activase 2, chloroplastic isoform X1 [*Phoenix dactylifera*], ribulose bisphosphate carboxylase/oxygenase activase 2, chloroplastic isoform X1 [*Phoenix dactylifera*], ribulose-1,5-bisphosphate carboxylase/oxygenase large subunit, partial (plastid) [*Torilis japonica*], ribulose-1,5-bisphosphate carboxylase/oxygenase, partial (chloroplast) [*Eriophorum viridicarinatum*], ribulose-1,5-bisphosphate carboxylase/oxygenase large subunit, partial (chloroplast) [*Eucalyptus stoatei*], ribulose-1,5-bisphosphate carboxylase/oxygenase large subunit [*Pimelea longiflora* subsp.* eyrei*], ribulose-1,5-bisphosphate carboxylase/oxygenase large subunit (RubisCO) [*Andreaea rothii*], and ribulose-1,5-bisphosphate carboxylase [*Euonymus bungeanus*]).
- ATP synthase subunit d, mitochondrial [*Phoenix dactylifera*] was found to be significantly changed only in drought stress with PEG and NaCl treated groups, whereas glucose-methanol-choline (GMC) oxidoreductase, putative [*Ricinus communis*] was found to be significantly changed only in drought stress either PEG or No Irrigation.
- 17 other proteins were significantly changed only in NaCl treated group (ribulose-1,5-bisphosphate carboxylase [*Moraea fugax*], chlorophyll a-b binding protein 6, chloroplastic [*Vitis vinifera*], phosphoglycerate kinase (PGK), cytosolic [*Glycine max*], ribulose-1,5-bisphosphate carboxylase/oxygenase large subunit [*Zantedeschia aethiopica*], ribulose-1,5-bisphosphate carboxylase, glyceraldehyde-3-phosphate dehydrogenase B (GPDB), chloroplastic [*Phoenix dactylifera*], dehydroascorbate (DHA) reductase-like [*Phoenix dactylifera*], serine acetyltransferase 1 (SAT1), chloroplastic-like [*Brassica napus*], ATP synthase beta subunit [*Toronia toru*], ATP synthase subunit beta, mitochondrial-like [*Phoenix dactylifera*], ATP synthase CF1 alpha chain [*Phoenix dactylifera*], beta-glucosidase 12-like [*Phoenix dactylifera*], chaperonin CPN60-2, mitochondrial-like [*Phoenix dactylifera*], chaperonin hsp60 [*Arabidopsis thaliana*], and uncharacterized protein LOC103705614 [*Phoenix dactylifera*]).
- Five proteins were significantly changed only under drought condition exerted by PEG (serine acetyltransferase 1, chloroplastic-like [*Brassica napus*], transketolase, chloroplastic [*Phoenix dactylifera*], ribulose-1,5-bisphosphate carboxylase/oxygenase large subunit [*Zantedeschia aethiopica*], oxygen-evolving enhancer protein 2, chloroplastic-like [*Phoenix dactylifera*], and cytochrome P450, putative [*Ricinus communis*]).
- Five more proteins were also significantly changed only under drought condition exerted by no irrigation (ribulose-1,5-bisphosphate carboxylase [*Euonymus bungeanus*], ribulose-1,5-bisphosphate carboxylase/oxygenase large subunit [*Zantedeschia aethiopica*], ribulose-bisphosphate carboxylase [*Lysichiton americanus*], ribulose-1,5-bisphosphate carboxylase/oxygenase large subunit [*Lunularia cruciata*], and oxygen-evolving enhancer protein 2, chloroplastic-like [*Phoenix dactylifera*]).

## 4. Discussion

This study described the response of 18-month-old date palm plants to severe salinity (48 g/L NaCl) and drought stress exerted by PEG (82.5 g/L) or no irrigation for one month as revealed by proteome analysis of leaves. Proteome analysis of roots and leaves revealed a synergetic responsive network under stress [12]. The DIGE analysis results showed that 55 protein spots were significantly changed as a result of salinity and drought stress. Salt stress showed 15 spots with higher protein abundance and 20 spots with lower protein abundance compared to control plants. Levels of ATP synthase CF1 alpha chain were significantly changed under all three stress conditions. Oxygen-evolving enhancer protein 2 was significantly changed as a result of salinity. Changes in the abundance of RubisCO activase and one of RubisCO fragments were significant in the same spots only for salt stress and drought stress with no irrigation. Jordan and Chollet [18] stated that the principal role of RubisCO activase is to release inhibitory sugar phosphates, such as ribulose-1,5-bisphosphate, from the active sites of RubisCO to allow its activation by CO_2_ through carbamylation, whereas Rokka et al. [19] reported that RubisCO activase functions as a chaperone during stress. In addition, Parker et al. [20] stated that the reduction of RubisCO activase due to exposure to NaCl might be the prime reason of declined photosynthetic activity under NaCl stress. RubisCO and its fragments were identified in three-month-old seedlings as a result of moderate salt and drought stress [11].

The current results indicate that the high concentration of NaCl has an inhibitory effect on date palm biosynthesis resulting in reduction in plant growth under high salinity. Mechanisms that contribute to date palm salt tolerance were described through miRNA-mediated gene expressions that are important for adaptation to salinity [21], whereas Carnavale Bottino et al. [22] ascribed salt tolerance in sugarcane to a number of miRNAs involved in salt stress responses in sugarcane.

Drought stress with PEG showed three spots with higher protein abundance and six spots with lower protein abundance compared to control plants. Golldack et al. [23] ascribed changes of the phosphorylation status of cellular proteins to abiotic stress such as drought or salinity. Reactive oxygen species function as an important regulator for many biological processes, such as stress responses, hormone signaling, cell growth, and development [24].

Drought stress with no irrigation did not show any spots with higher protein abundance and showed 19 spots with lower protein abundance compared to control plants. Six spots showed lower protein abundance in salt stress and drought stress due to no irrigation. Only one spot showed lower protein abundance in all three studied stresses. Drought stress primarily results in a reduced rate of photosynthesis [6, 15, 16]. Plant leaf proteome analysis supported this by the lower protein abundance of most photosynthesis enzymes such as ribulose-1,5-bisphosphate carboxylase/oxygenase, oxygen-evolving enhancer protein 2, chloroplastic-like, and cytochrome P450, putative [11]. These findings are supporting those of Loreto et al. [25] who stated that the activity of the photosynthetic electron transport chain is finely tuned to the availability of CO_2_, and photosystem II activities often decline in parallel by drought stress. In addition, Bota et al. [26] reported that the very severe drought conditions lead to stomatal closures that limit photosynthesis due to a decline in RubisCO activity.

## 5. Conclusion

The proteome analysis of date palm under salinity and drought conditions indicated that both salinity and drought tolerance genes were differentially expressed resulting in high or low protein abundance of the chosen protein spots as a result of exposure to drought and salinity stress condition. In addition, drought stress due to no irrigation caused downexpression of all genes controlling drought tolerance.

## Supplementary Material

MS analysis of the significantly changed spots and their behavior in other studied stresses.

## Figures and Tables

**Table 1:** Labeling scheme for 8 gels: the internal standard (IS) and each analyzed sample were labeled with CyDye 2, 3, or 5 as shown in the table. The internal standard is generally the mixture of the same portions of all analyzed samples.

Gel number	CyDye 2	CyDye 3	CyDye 5
1	IS	88”	51^*∗*^
2	IS	61^∧^	52^*∗*^
3	IS	54^*∗*^	76^#^
4	IS	55^*∗*^	86”
5	IS	77^#^	62^∧^
6	IS	89”	64^∧^
7	IS	63^∧^	90”
8	IS	80^#^	78^#^

*∗*: control, #: PEG drought stress, ∧: no irrigation drought stress, and ”: salt stress.

**Table 2:** DIGE results: overview of the significantly changed spots and their behavior in other studied stresses.

Spot number	NaCl/control		PEG/control		Irrigation/control		Enzyme abbreviation
	*t*-test	Av. ratio	*t*-test	Av. ratio	*t*-test	Av. ratio	
65	0.0082^*∗*^	2.17^∧^	0.37	1.2	0.084	1.9	No protein hit
67	0.02^*∗*^	2.24^∧^	0.79	1.03	0.13	1.65	No protein hit
69	0.025^*∗*^	2.44^∧^	0.82	−1.11	0.31	1.58	LOC103705614
299	0.025^*∗*^	2.4^∧^	0.5	−1.32		2.6	No protein hit
323	0.011^*∗*^	2.41^∧^	0.44	1.2	0.15	1.74	CPhsp60
337	0.047^*∗*^	2.16^∧^	0.65	−1.35	0.54	1.62	ATPase CF1 alpha
348	0.019^*∗*^	3.32^∧^	0.64	1.06	0.37	1.65	CPN60-2
373	0.0011^*∗*^	2^∧^	0.44	−1.11	0.28	1.72	No protein hit
383	0.0043^*∗*^	2.42^∧^	0.46	1.36	0.31	1.36	SATase1
402	0.034^*∗*^	2.47^∧^	0.83	−1.16	0.73	1.11	Beta-glucosidase
403	0.02^*∗*^	2.71^∧^	0.77	1.14	0.63	1.18	ATPase CF1 alpha
485	0.03^*∗*^	2.14^∧^	0.69	1.12	0.087	2.1	ATPase beta
497	0.0053^*∗*^	2.25^∧^	0.9	−1.02	0.059	2.09	ATPase beta
529		8.29	0.05^*∗*^	−3.57^*∗*^		−2.5	RubisCO
731	0.019^*∗*^	2.25^∧^	0.59	−1.18	0.093	1.79	SAT1
967		−2.33	0.026^*∗*^	−2.6^*∗*^	0.26	−1.7	CYP450
988	0.047^*∗*^	−2.52^*∗*^	0.061	−1.72	0.36	−1.31	GPDB
1021	0.91	−1	0.04^*∗*^	−2.28^*∗*^	0.9	−1.04	No protein hit
1119^#^	0.011^*∗*^	−2.1^*∗*^	0.19	−1.21	0.0031^*∗*^	−2.72^*∗*^	RubisCO activase 2
1126^#^	0.037^*∗*^	−2.65^*∗*^	0.63	−1.2	0.031^*∗*^	−2.23^*∗*^	RubisCO activase 2
1270	0.44	−1.21	0.093	−1.58	0.011^*∗*^	−2.22^*∗*^	No protein hit
1338	0.06	−1.39	0.25	1.15	0.0086^*∗*^	−2.04^*∗*^	No protein hit
1417	0.015^*∗*^	−2.1^*∗*^	0.49	−1.23	0.077	−1.7	No protein hit
1427”	0.0033^*∗*^	−2.61^*∗*^	0.00043^*∗*^	−2.4	0.025^*∗*^	−2.29^*∗*^	ATPase CF1 alpha
1433	0.026^*∗*^	−2.28^*∗*^	0.1	−1.45	0.12	−1.59	No protein hit
1471	0.61	−1.1	0.45	−1.16	0.023^*∗*^	−2.01^*∗*^	No protein hit
1485	0.038^*∗*^	2.37^∧^	0.88	1.07	0.71	1.11	RubisCO
1520	0.69	−1.06	0.74	1.06	0.007^*∗*^	−2.03^*∗*^	No MS result
2070	0.16	−1.51	0.93	1.06	0.033^*∗*^	−2.59^*∗*^	OEEP2
2117	0.0055^*∗*^	−2.12^*∗*^	0.1	−1.26	0.014	−1.67	RubisCO
2177	0.23	−1.52	0.034	−1.99	0.024^*∗*^	−2.42^*∗*^	GMC oxidoreductase
2201	0.058	−2.06	0.044^*∗*^	−2.34^*∗*^	0.062	−1.53	OEEP2
2202	0.03^*∗*^	−2.17^*∗*^	0.2	−1.23	0.022	−1.7	RubisCO
2222	0.57	−1.08	0.9	1.02	0.0067^*∗*^	−2.1^*∗*^	RubisCO
2267	0.0071^*∗*^	−2.09^*∗*^	0.009	−1.82	0.2	−1.43	ATPase d
2440		1.07	0.048^*∗*^	2.31^∧^	0.67	1.31	RubisCO
2497	0.028^*∗*^	−2.14^*∗*^	0.24	−1.36	0.035	−1.85	RubisCO
2526^#^	0.027^*∗*^	−2.35^*∗*^	0.33	−1.41	0.0073^*∗*^	−3.11^*∗*^	RubisCO
2553	0.74	1.17	0.23	−1.39	0.032^*∗*^	−2.09^*∗*^	No protein hit
2566	0.04	−1.95	0.022	−1.98	0.0044^*∗*^	−2.63^*∗*^	EFTuA
2568	0.062	−1.54	0.3	−1.29	0.0074^*∗*^	−2.49^*∗*^	RubisCO
2569	0.0075^*∗*^	−2.04^*∗*^	0.046	−1.46	0.0047	−1.98	No protein hit
2589^#^	0.023^*∗*^	−2.27^*∗*^	0.2	−1.37	0.0049^*∗*^	−2.45^*∗*^	RubisCO
2602	0.58	1.22	0.0078^*∗*^	2.5^∧^	0.061	1.44	No MS result
2620^#^	0.014^*∗*^	−2.74^*∗*^	0.022	−1.95	0.0043^*∗*^	−2.55^*∗*^	RubisCO
2636	0.028^*∗*^	−2.28^*∗*^	0.74	−1.09	0.32	−1.34	PGK
2655	0.022^*∗*^	−2.34^*∗*^	0.18	−1.46	0.37	−1.29	CBP6
2668	0.022^*∗*^	−2.13^*∗*^	0.015	−1.61	0.028	−1.71	OEEP1
2673	0.48	1.63	0.028^*∗*^	2.03^∧^	0.12	1.74	TKL
2680	0.14	−1.75	0.93	1.02	0.035^*∗*^	−2.25^*∗*^	RubisCO
2695	0.15	−1.36	0.98	1.06	0.021^*∗*^	−2.36^*∗*^	RubisCO
2696^#^	0.035^*∗*^	−2.3^*∗*^	0.2	−1.42	0.041^*∗*^	−2.18^*∗*^	RubisCO
2720	0.039^*∗*^	−3.49^*∗*^	0.24	−1.91	0.059	−2.81	RubisCO
2790	0.08	−1.84	0.025^*∗*^	−2.38^*∗*^	0.25	−1.61	SATase1
2808	0.03^*∗*^	−2.62^*∗*^	0.32	−1.35	0.23	−1.67	No protein hit

*∗* indicates for *t*-test *p* value lower than 0.05; for average ratio it indicates Av. ratio < −2; ∧: Av. ratio > 2; # indicates the spot numbers common for 2 studied stresses; ”: 1 marked spot was common for all three stress treatments.
