# Supplementary material for: Proteome Analysis of Date Palm (Phoenix dactylifera L.) under Severe Drought and Salt Stress
Source: Int J Genomics. 2016 Oct 20;2016:7840759. doi: 10.1155/2016/7840759 (PMC5093262; doi:10.1155/2016/7840759)
Supplement: Supplementary file 2 [file 7840759.f2.doc]

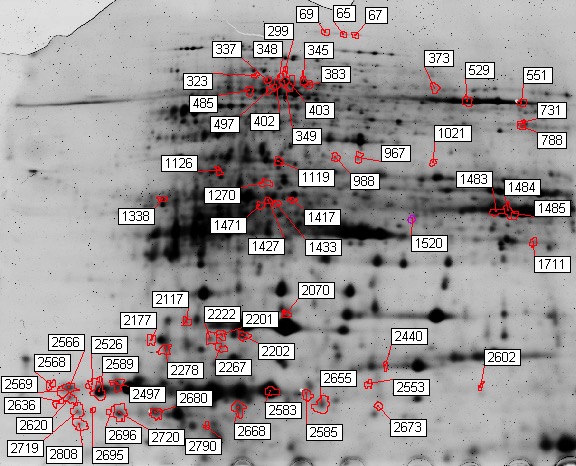


Supplementary FIGURE 5: Preparative gel P2: spots cut for MS analysis. All picked spots are highlighted with red spot contours and number boxes.
